# Cost‐Effectiveness Analysis of the Milan System for Reporting Salivary Gland Cytopathology in Fine‐Needle Aspiration Cytology of Salivary Gland Lesions

**DOI:** 10.1002/cam4.71579

**Published:** 2026-02-08

**Authors:** Louis Jansen, Lisa Nachtsheim, Sofia Kourou, Philipp Wolber, Kariem Sharaf, Kevin Hansen, Sami Shabli, Julia van de Loo, Alexander Quaas, Christoph Arolt, Marianne Engels, Lena Hieggelke, Luc G. T. Morris, Jens Peter Klussmann, Marcel Mayer

**Affiliations:** ^1^ Department of Otorhinolaryngology, Head and Neck Surgery, Medical Faculty and University Hospital Cologne University of Cologne Cologne Germany; ^2^ Center for Molecular Medicine Cologne (CMMC) University of Cologne Cologne Germany; ^3^ Head and Neck Service and Immunogenomic Oncology Platform, Department of Surgery Memorial Sloan Kettering Cancer Center New York New York USA; ^4^ University of Cologne Institute for General Pathology and Pathologic Anatomy, Medical Faculty and University Hospital Cologne Cologne Germany

**Keywords:** fine needle aspiration cytology, head and neck oncology, health economics, salivary gland cancer, salivary gland tumor

## Abstract

**Introduction:**

Salivary gland lesions (SGL) are a rare and heterogeneous group of benign and malignant masses. Fine‐needle aspiration cytology (FNAC), guided by the Milan System for Reporting Salivary Gland Cytopathology (MSRSGC), offers a minimally invasive method for early differentiation of SGL. The purpose of this study was to evaluate the cost‐effectiveness of FNAC in diagnosing major SGL within the MSRSGC framework.

**Methods:**

Three decision tree models were created based on probabilities from real‐world and literature data. Real‐world data was derived from the previously published largest single‐center study evaluating FNAC performance of SGL to date. Costs were determined from German and American fee catalogs. Multiple Monte Carlo simulations were run to assess the cost‐effectiveness of performing FNAC within the MSRSGC framework under different conditions for both health care systems.

**Results:**

Using decision analysis, FNAC followed by surgery, if indicated, was less costly than upfront surgery. The cost reduction through FNAC was over 30% for all models. Cost reduction per case through FNAC followed by surgery, if indicated, compared to upfront surgery ranged between $5606 and $13,096 in the US model (average costs for upfront surgery: $17,472) and between 2465€ and 5337€ in the German model (average costs for upfront surgery: 8018€). When enhancing the German model with real world data, the cost reduction ranged between 2478€ and 5954€ (average costs for upfront surgery: 7988€).

**Conclusion:**

In this model based on MSRSGC estimates and real‐world data, FNAC followed by surgery, if indicated, proved to be a more cost‐efficient approach to diagnosing SGL than upfront surgery. Thus, patients and healthcare systems benefit from high‐output centers that guarantee expert cytopathological diagnosis.

## Graphical abstract

This study evaluated the cost‐effectiveness of fine‐needle aspiration cytology (FNAC), guided by the Milan System, for diagnosing salivary gland lesions. Decision tree models using real‐world and literature data showed that FNAC followed by surgery, when indicated, reduced costs by 30% or more in both U.S. and German healthcare models compared to upfront surgery. Overall, FNAC proved to be a more cost‐efficient strategy, highlighting the value of expert cytopathology in high‐volume centers.

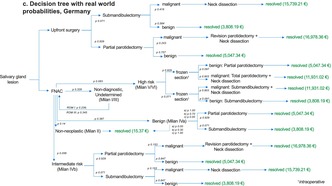

## 1 Introduction

Salivary gland lesions (SGL) encompass a rare and diverse group of neoplasms and diseases, accounting for approximately 3%–6% of all head and neck tumors [1, 2]. The majority of SGL are salivary gland tumors (SGT), with recent studies reporting that between 70% and 80% are benign and between 20% and 30% are malignant [3, 4, 5]. Globally, the annual incidence of SGT is estimated at 2.5–3.0 per 100,000 cases [6]. Pleomorphic adenoma (70%) and Warthin's tumor (17%) are the most frequent benign tumors [3, 7]. The frequency of malignant lesions is more heterogeneous and depends heavily on the subsites that were studied [3]. Despite their rarity, SGL pose diagnostic and management challenges due to their histopathological diversity and the critical need to distinguish benign from malignant lesions early in the clinical pathway [1, 8, 9].

Fine‐needle aspiration cytology (FNAC) has been established as a pivotal, minimally invasive diagnostic tool for evaluating SGT, offering a rapid and reliable assessment that can substantially influence patient management [1, 6, 10]. The ability to preoperatively differentiate benign from malignant lesions is crucial for optimizing surgical planning, reducing unnecessary procedures, and conserving healthcare resources [10, 11].

The introduction of the Milan System for Reporting Salivary Gland Cytopathology (MSRSGC) has further advanced the standardization and clinical relevance of FNAC [12]. This system stratifies SGL into six diagnostic categories—non‐diagnostic, non‐neoplastic, atypia of undetermined significance (AUS), benign neoplasm, salivary gland neoplasm of uncertain malignant potential (SUMP), suspicious for malignancy, and malignant—each with a defined risk of malignancy (ROM) [10, 12]. By reducing diagnostic uncertainty and encouraging risk‐informed decision‐making, the MSRSGC enhances diagnostic reliability, aims to reduce the number of surgeries required, thereby reducing complication rates, and may also contribute to a more efficient use of healthcare resources.

This study provides a critical appraisal of the cost‐effectiveness of FNAC for major SGL. By contextualizing these findings within the structured framework of the MSRSGC, we aimed to assess the economic benefits of adopting standardized cytological assessment.

## 2 Methods

### 2.1 Real‐World Data

Real‐world data used in this study was derived from a previously published study evaluating the performance of FNAC of SGL from the Department of Otolaryngology, Head and Neck Surgery at University Hospital Cologne in Germany [6]. The probabilities and distributions used in Figure and Model 1c are derived from that study. In the study by Mayer et al., all examined cases with final histopathological diagnosis (*n* = 1289) were classified according to the MSRSGC and followed a standardized clinical and pathological protocol.

### 2.2 Decision Tree

**FIGURE 1:**
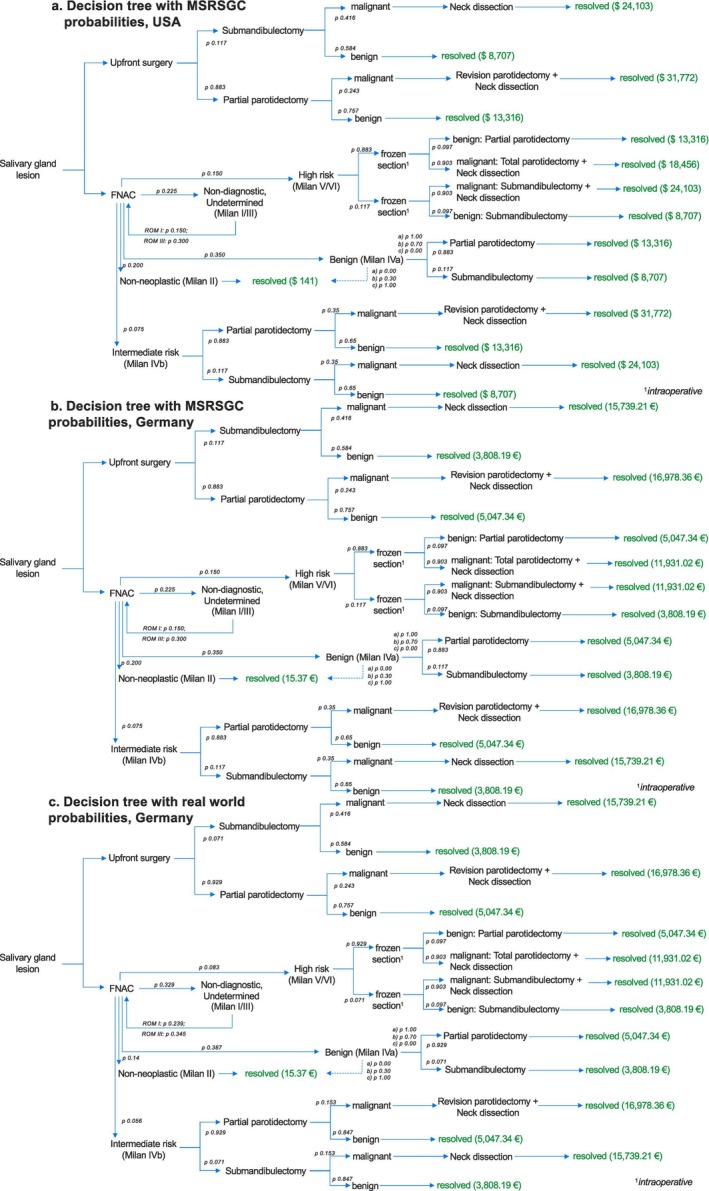
Decision tree modeling costs of initial FNAC versus upfront surgery for a patient with an ultrasound‐confirmed parotid or submandibular salivary gland nodule. Each branching point represents a decision node. End nodes are color‐coded in green. Probabilities shown at applicable nodes. Model (1a) MSRSGC estimates, United States; Model (1b) MSRSGC estimates, Germany; Model (1c) real world data from University Hospital Cologne, Germany (2025). FNAC, fine‐needle aspiration cytology; MSRSGC, Milan reporting system for salivary gland cytopathology; *p*, probability.

A decision tree was developed to model and evaluate the clinical and economic outcomes of possible diagnostic and treatment strategies. Overall, three different models were simulated. The decision tree was modified to simulate costs within the US and German healthcare systems according to MSRSGC estimates and additionally to simulate costs within the German healthcare system according to real‐world data collected in Germany (Figure 1a–c) [6, 12]. The decisive primary node involved selecting whether to perform FNAC or upfront surgery. Further decision nodes were dependent on pathologic results and resulted in distinct subsequent diagnostic and therapeutic pathways recommended by the MSRSGC. Notably, intraoperative frozen section was part of the decision tree for all lesions classified as MSRSGC category V/VI (suspicious for malignancy/malignant) to confirm the FNAC diagnosis. Moreover, in line with the MSRSGC recommendations, MSRSGC category I and III lesions were subject to one repeat FNAC in our models [12]. If they were again reclassified as category I or III lesions on their second run‐through, they were subjected to immediate surgery (partial parotidectomy/submandibulectomy). In case of malignancy, they entered the revision surgery arm. Probabilities at each decision node, including the rates of benign and malignant findings, as well as subsequent surgical outcomes, were assigned based on published epidemiological data or real‐world clinical data (Table 2).

### 2.3 Probabilities, Cost Estimates, and Complication Rates

US cost estimates for selected procedures were obtained from the Medicare Physician Fee Schedule 2025 and the FAIR Health consumer database [13, 14]. German cost estimates were calculated using an established and validated Diagnosis Related Groups (DRG) web grouper for selected operation and procedure classification system/“Operationen‐ und Prozedurenschlüssel” (OPS) codes with an average length of hospital stay [15]. The costs for FNAC were derived from the German uniform evaluation standard/“Einheitlicher Bewertungsmaßstab” (EBM) catalog (Table 1) [16]. Discounting was not accounted for within this analysis. The probabilities and distributions for the simulation models and complication rates were derived through a literature review from published data and the official MRSSGC 2nd edition (Tables 2 and 3) [3, 12, 17, 18, 19, 20, 21, 22]. Complication rates were gathered separately through a literature review to prevent the models from overfitting while simultaneously accounting for the heterogenous nature of salivary gland surgeries in different settings (Table 3). All parameters, including probabilities, costs, and outcomes, were reviewed by clinical subject‐matter experts to ensure medical plausibility. Currency conversions between euros and U.S. dollars were not performed in this study due to continuously fluctuating exchange rates. Therefore, the reported cost estimates should be converted to other currencies at the time of manuscript evaluation as appropriate.

**TABLE 1:** Approximated costs of analyzed procedures in the United States and Germany, 2025.

Procedure	CPT code	Cost ($)	OPS/EBM code	Cost (€)
Partial parotidectomy	42420	13,316	5‐262.0	5047.34
Submandibulectomy	42440	8707	5‐262.4	3808.19
Total parotidectomy + ND	42426	18,456	5‐262.1, 5‐403.20	11,931.02
Submandibulectomy + ND	42440, 38724	24,103	5‐262.4, 5‐403.20	11,931.02
ND	38724	15,396	5‐403.20	9864.74
FNAC (US guided)	10022	141	02340+33011	15.37

Abbreviations: CPT, current procedural terminology; EBM, “Einheitlicher Bewertungsmaßstab”; FNAC, fine‐needle aspiration cytology; ND, neck dissection; OPS, “Operationen‐ und Prozedurenschlüssel”; US, ultrasound.

**TABLE 2:** Outcome probabilities of examined procedures according to real‐world data from the University Hospital Cologne, Germany and MRSSGC data.

MSRSGC category	RWD (*n* = 1289)		MSRSGC		MSRSGC recommendation
	*n* (%)	ROM (%)	(%)	ROM (%)	
Non‐diagnostic, I	314 (24.2)	23.9	10–20	15	Repeat FNAC/Clinical and radiological correlation
Non‐neoplastic, II	182 (14.0)	4.4	15–25	11	Clinical follow up/radiological correlation
AUS, III	113 (8.7)	34.5	< 10	30	Repeat FNAC/Surgery
NP‐benign, IVa	501 (38.7)	1.0	30–40	< 3	Surgery/Clinical and radiological follow up
NP‐SUMP, IVb	72 (5.6)	15.3	< 10	35	Surgery
SFM, V	54 (4.2)	74.1	< 5	83	Surgery
Malignant, VI	53 (4.1)	96.2	10–15	> 98	Surgery

Abbreviations: AUS, atypia of undetermined significance; FNAC, fine‐needle aspiration cytology; MSRSGC, Milan Reporting System for Salivary Gland Cytopathology; NP, neoplasm; ROM, risk of malignancy; RWD, real‐world data; SFM, Suspicious for malignancy; SUMP, salivary gland neoplasm of uncertain malignant potential.

**TABLE 3:** Approximated complication estimates for selected surgical procedures.

Procedure	Complication rates [%]	Source
FNAC (ultrasound‐guided)	< 1	[22]
Partial parotidectomy	3.5	[18]
Submandibulectomy	3.4–7.7	[19]
Total parotidectomy + neck dissection	13.9	[18]
Submandibulectomy + neck dissection	11–52	[18, 19, 21]
Neck dissection	11–52	[18, 21]

Abbreviation: FNAC, fine‐needle aspiration cytology.

### 2.4 Statistical Analysis

Risk of malignancy (ROM) for real‐world data from the University Hospital Cologne, Germany as well as the 2nd edition of the MSRSGC are depicted in Table 2 [6, 12]. Monte Carlo simulations (MCS) for *n* = 10,000 cases were carried out utilizing probabilities and distributions from the decision tree (Figure 1). This resulted in three statistical models named 1a, b, and c according to the decision trees depicted in Figure 1a–c.

To reflect the heterogenous management of MSRSGC category IVa lesions, each of the three models was run three times with adjustments for the surgery rates of MSRSGC category IVa lesions (benign). The base model accounted for 100% of all MSRSGC category IVa lesions receiving surgery, while the two alternate versions accounted for 70% and 0% of all MSRSGC Iva lesions receiving surgery. The share of 70% was set to account for benign neoplasms that have shown tendencies to transition to malignant tumors in previous studies. These tumors included pleomorphic adenomas and basal cell adenomas. Risk of malignancy estimates for the aforementioned benign tumors were derived from nationwide epidemiologic studies [7, 23, 24]. Statistical analysis was carried out using the R Statistical software version 4.4.1 and RStudio version 2024.12.1, Build 563 including the R packages *dplyr* and *base* [25]. GraphPad Prism Version 10.3.1 was used to visualize results (GraphPad Software Inc., Boston, USA) [26].

## 3 Results

### 3.1 Model 1a—MSRSGC Estimates (USA)

**FIGURE 2:**
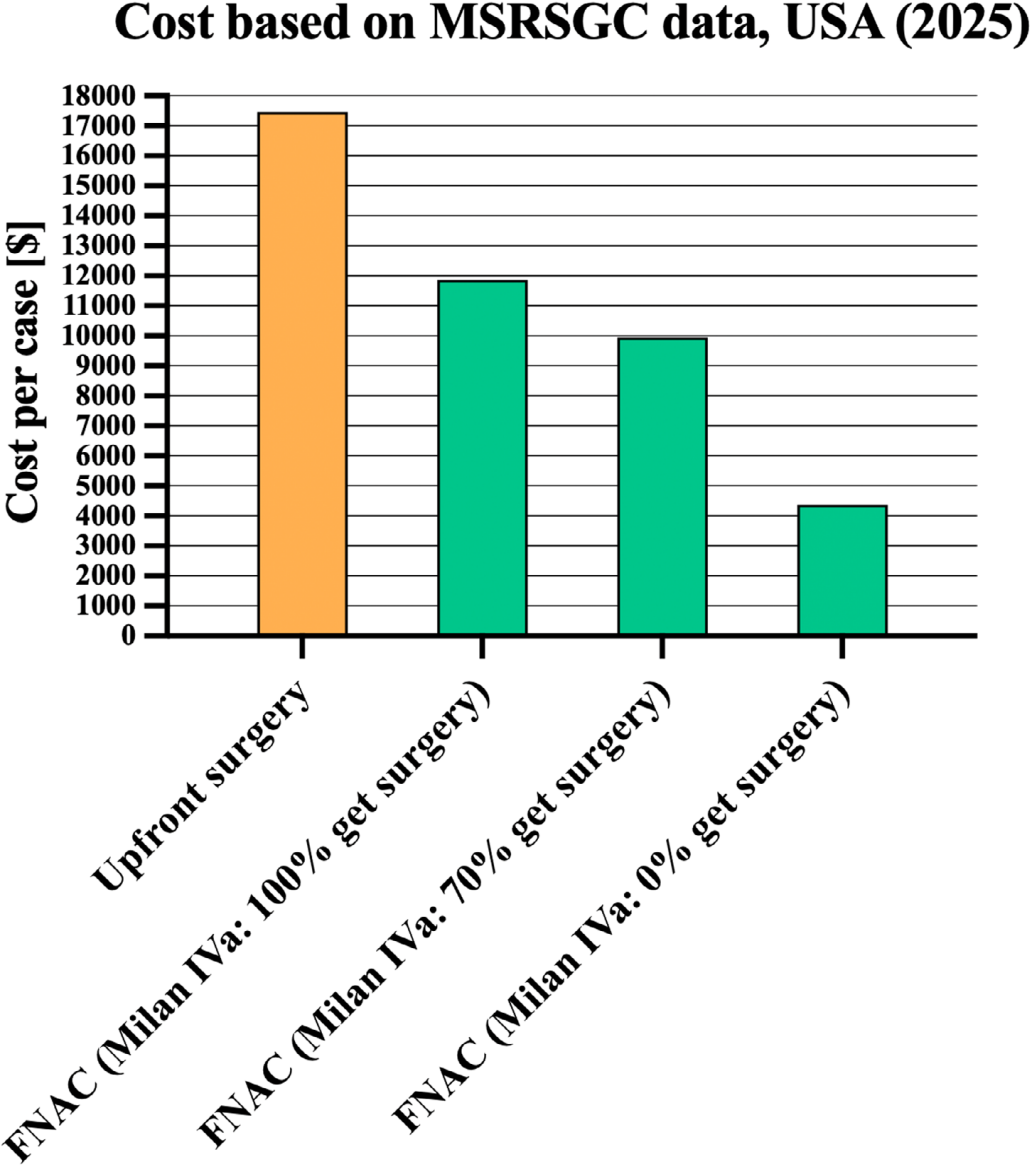
Monte Carlo simulation cost based on MSRSGC data (Model 1a) dependent on therapeutic approach for US healthcare system (2025). FNAC, fine‐needle aspiration cytology; MSRSGC, Milan Reporting System for Salivary Gland Cytopathology.

MCS for *n* = 10,000 cases from the US showed an average cost of $17,472 per case for upfront surgery. In comparison, the cost of FNAC followed by surgery, if indicated, was $11,866 when all MRSSGC category IVa cases received surgery. It decreased to $9947 when only 70% were operated on, and to $4377 when all MRSSGC category IVa cases were treated conservatively (Figure 2). This corresponds to a cost reduction of FNAC followed by surgery, if indicated, compared to upfront surgery of 32%, 43%, and 74%, respectively.

### 3.2 Model 1b—MSRSGC Estimates (Germany)

**FIGURE 3:**
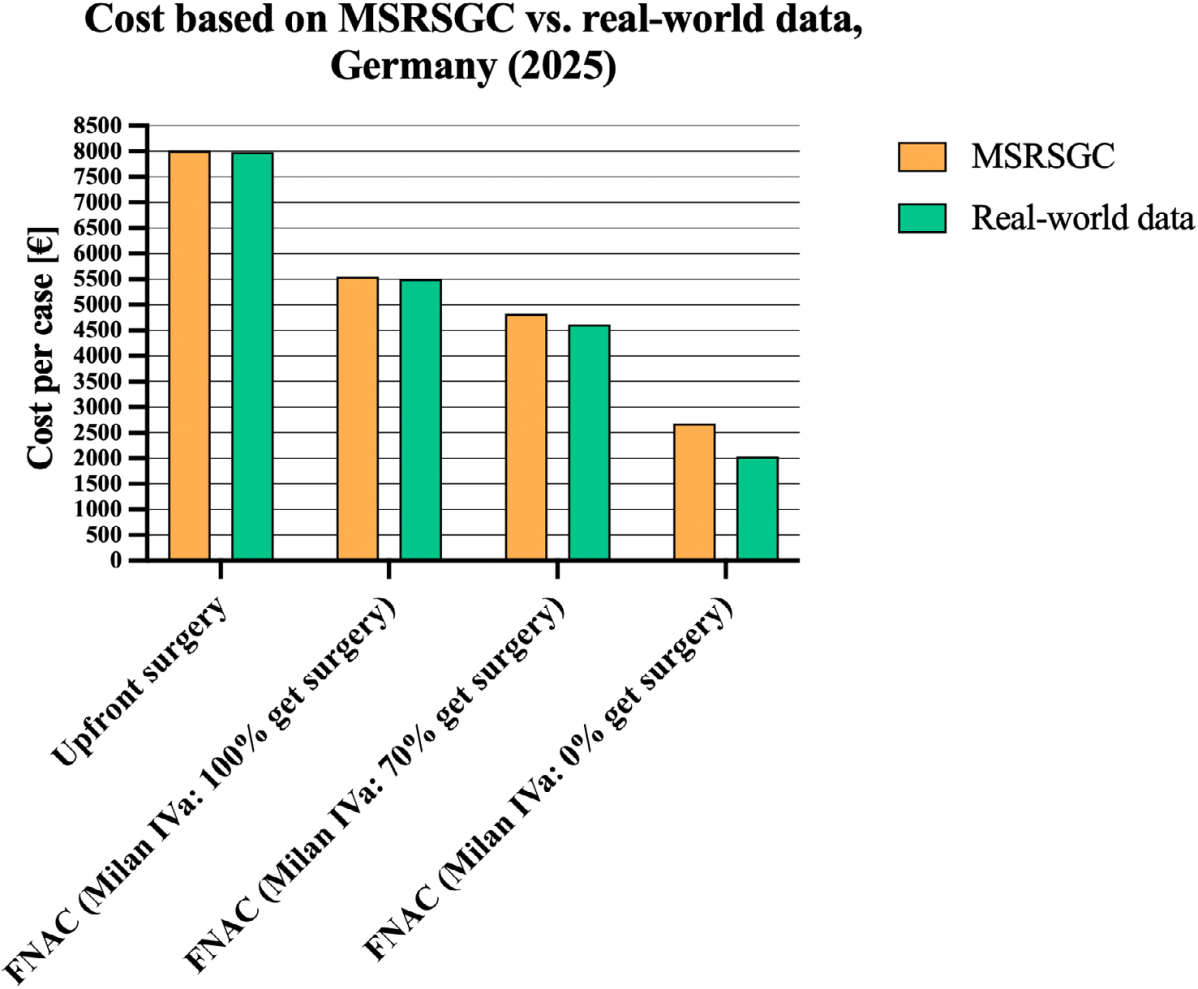
Monte Carlo simulation cost based on MSRSGC (Model 1b) and real‐world data (Model 1c) dependent on therapeutic approach for the German healthcare system (2025). FNAC, fine‐needle aspiration cytology; MSRSGC, Milan Reporting System for Salivary Gland Cytopathology.

MCS for *n* = 10,000 cases from Germany showed an average cost of 8018€ per case for upfront surgery. Compared to that, the average cost of FNAC followed by surgery, if indicated, was 5553€ when all MRSSGC category IVa cases received surgery. It decreased to 4825€ when only 70% were operated on, and to 2681€ when all MRSSGC category IVa cases were treated conservatively (Figure 3). This corresponds to a cost reduction of FNAC followed by surgery, if indicated, compared to upfront surgery of 30%, 39%, and 66%, respectively.

### 3.3 Model 1c—Real World Data Estimates (Germany)

The MCS based on real‐world data from the University Hospital Cologne, Germany for *n* = 10,000 cases showed an average cost of 7988€ per case for upfront surgery. The cost of FNAC followed by surgery, if indicated, was 5510€ when all MRSSGC category IVa cases received surgery. It was 4618€ when only 70% were operated on, and 2034€ when all MRSSGC category IVa cases were treated conservatively (Figure 3). This corresponds to a cost reduction of FNAC followed by surgery, if indicated, compared to upfront surgery of 31%, 42%, and 74%, respectively.

## 4 Complications

The previously published complication rates of the different therapeutic approaches are listed in Table 3. FNAC is the procedure with the lowest complication rate. Severe complications like permanent facial palsy were found in 3.5% of all partial parotidectomies and in up to 7.7% of all submandibulectomies [18, 19]. The procedure associated with the highest rate of complications was neck dissection. However, the complication rates encompassed a wide range of complications that were dependent on the patient's history and previous treatments. Previous radiotherapy and chemotherapy increased complication rates, as well as previous surgeries [18, 20, 21].

## 5 Discussion

This study is the first to demonstrate that FNAC within the MSRSGC framework is a cost‐effective diagnostic approach for SGL. The decision analysis revealed clear economic benefits for both the US and German healthcare systems, highlighting the ability of FNAC to reduce the associated costs and complications.

From a health economics perspective, our analysis is consistent with prior literature showing that upfront surgeries, particularly if performed without clear cytological indication, lead to increased resource utilization and higher aggregate healthcare spending [11, 27]. A study by Layfield et al. published in 2006 represented the first analysis of the cost‐effectiveness of FNAC in the evaluation of SGL [27]. Despite its pioneering role, it is 19 years old and employs a more simplistic decision‐tree approach compared to the present study. Importantly, the study by Layfield et al. does not incorporate the MSRSGC, which has since become integral for risk stratification and management decisions in salivary gland FNAC [6, 12]. As such, while foundational, its findings do not align with current clinical standards and do not encompass the nuanced classification systems used today.

Our models approach resulted in cost savings of over 30% in both US and German models. The effect was even more pronounced when conservative management was applied to MSRSGC category IVa lesions, as demonstrated in both simulated and real‐world cohorts. Benign SGL classified as MSRSGC category IVa carry a very low risk of malignancy of < 3% [12]. Real‐world data from the University Hospital Cologne, Germany has even shown rates as low as 1% [6]. In case of a MSRSGC category IVa lesion, the MSRSGC recommends that management may include either surgical excision or clinical and radiological follow‐up [12]. Surgery is often advised because, while these tumors are benign, they may cause discomfort and undergo malignant transformation if left untreated [7, 23, 28]. Malignant transformation is predominantly found in pleomorphic adenoma and basal cell adenoma with 5%–15% and 4% respectively [24, 29]. Other entities, including Warthin's tumor and lymphadenoma, show sporadic malignant transformation rates well below 1% [30, 31]. Especially for the latter, observation may be appropriate in select cases, particularly when surgery poses higher risk due to patient comorbidities or frailty, and if the lesion is stable and asymptomatic.

In line with the MSRSGC recommendations, MSRSGC category I and III lesions were subject to one repeat FNAC in our model. Another suitable approach would be to continue with surgery after the first inconclusive FNAC, which is also covered by the MSRSGC recommendations for MSRSGC category III lesions [12]. We opted for one repeat FNAC for both MSRSGC category I and III lesions in our models as it is an almost risk‐free and cheap procedure. Moreover, patients may avoid unnecessary or revision surgery in case the second FNAC yields Milan II, IVa, V, or VI results [22]. Core needle biopsy (CNB) presents an alternative to repeat FNAC as it yields fewer nondiagnostic samples and a higher ability to call malignancy and provide a specific entity diagnosis than FNAC [10, 32]. In our models we opted for an FNAC‐only approach as the MSRSGC was developed for FNAC and is endorsed in this role by the American Society of Clinical Oncology for FNAC reporting and risk stratification; it is not a validated or guideline‐endorsed system for CNB histology reporting [10].

Beyond direct costs, FNAC as a first‐line diagnostic modality demonstrates the lowest complication rate among procedures examined (< 1%), compared with higher risks for surgical interventions like partial parotidectomy or submandibulectomy [18, 19, 22]. Notably, severe complications such as permanent facial nerve palsy are restricted to major surgical procedures and are not observed with FNAC [22]. These findings from previous studies support the safety of FNAC and its role as the diagnostic measure of choice. Complications were not included in our MCS models as the rates differ widely depending on the heterogenous nature of the patient collectives. Also, we wanted to prevent our models from overfitting. Overfitting arises when models are too complex relative to the amount or nature of the data, introducing high variance and poor predictive performance on new data [33]. Importantly, the management of complications is associated with additional costs. Since FNAC may reduce overtreatment and thus the number of surgeries, it can be assumed that the real cost gap between a strategy of FNAC followed by surgery, if indicated, and upfront surgery is even larger when considering the high costs that arise from treatment of complications.

Another important uncertainty of this analysis is the heterogenous nature of revision and cancer surgery. The costs for those surgeries could be underestimated due to unmodeled factors, such as nerve reconstruction, free flap reconstruction, or mastoidectomy [18, 34, 35]. However, the overall economic advantage of FNAC appears robust, since we suspect that revision surgeries would predominantly become necessary in the upfront surgery arm, further widening the cost gap. It has to be noted that revision practices among cancer centers differ as decision‐making should balance oncologic benefit against facial nerve morbidity, integrate margin status, grade, T/N stage, and patient factors, and may involve imaging restaging and multidisciplinary planning [10, 36].

A key limitation of this study is that, while MCS are powerful tools for modeling uncertainty and exploring possible outcomes, they are often unable to fully capture the heterogeneous nature of real‐world patient populations and clinical practices [37, 38]. This holds particularly true for preexisting health conditions and varying degrees of frailty. These simulations frequently rely on assumptions of population and treatment homogeneity or simplified input distributions, which may not accurately represent the spectrum of comorbidities and physiological differences found in actual patient collectives [38]. A step towards more accurate results was our supplementation of real‐world data and the comparison to MSRSGC estimates. However, variations in risk profiles and treatment responses may still limit the generalizability of the study's findings.

Also, the cost estimates, especially for the US healthcare system, are estimates that do not reflect the heterogeneity of US insurance providers. Costs may significantly differ from our estimates depending on the location and practitioner [39]. Among the other named limitations, this cost variety may also compromise the generalizability of our model.

Conversely, the strength of this study is that it is the first to examine the cost‐effectiveness of FNAC within the MSRSGC framework. By enhancing our models with real‐world data, the results support that institutions and healthcare systems should favor FNAC as the primary diagnostic pathway, ensuring that cytological risk stratification informs surgical triage, conserves resources, and minimizes patient morbidity [6]. Future studies may further refine cost models by including long‐term outcomes and indirect costs, but present evidence strongly supports routine FNAC with MSRSGC reporting in clinical practice in terms of cost‐effectiveness aspects.

## 6 Conclusion

In conclusion, this study demonstrates that FNAC followed by surgery, if indicated, is a cost‐effective diagnostic tool for SGL compared to upfront surgery and may also aid in reducing costs for surgical complications. The results confirmed the cost‐effectiveness of FNAC through a real‐world data‐enhanced Monte Carlo simulation. These findings underscore the importance of expert pathological assessment of FNAC of SGL within the framework of the MSRSGC, as this improves patient outcomes and allocates healthcare assets more efficiently.

## Author Contributions

**Louis Jansen:** conceptualization (equal), formal analysis (equal), funding acquisition (equal), investigation (equal), methodology (equal), software (equal), visualization (equal), writing – original draft (equal), writing – review and editing (equal). **Lisa Nachtsheim:** supervision (equal), writing – review and editing (equal). **Sofia Kourou:** visualization (equal), writing – review and editing (equal). **Philipp Wolber:** supervision (equal), writing – review and editing (equal). **Kariem Sharaf:** supervision (equal), writing – review and editing (equal). **Kevin Hansen:** supervision (equal), writing – review and editing (equal). **Sami Shabli:** data curation (equal), resources (equal), supervision (equal), writing – review and editing (equal). **Julia van de Loo:** visualization (equal), writing – review and editing (equal). **Alexander Quaas:** formal analysis (equal), methodology (equal), supervision (equal), writing – review and editing (equal). **Christoph Arolt:** formal analysis (equal), methodology (equal), supervision (equal), writing – review and editing (equal). **Marianne Engels:** formal analysis (equal), methodology (equal), supervision (equal), writing – review and editing (equal). **Lena Hieggelke:** formal analysis (equal), methodology (equal), supervision (equal), writing – review and editing (equal). **Luc G. T. Morris:** formal analysis (equal), methodology (equal), supervision (equal), writing – review and editing (equal). **Jens Peter Klussmann:** conceptualization (equal), funding acquisition (equal), methodology (equal), supervision (equal), writing – review and editing (equal). **Marcel Mayer:** conceptualization (equal), data curation (equal), formal analysis (equal), funding acquisition (equal), methodology (equal), supervision (equal), writing – original draft (equal), writing – review and editing (equal).

## Funding

Marcel Mayer is currently sponsored by the German Research Foundation (Grant 539250008) and the Jean Uhrmacher Foundation. Louis Jansen is currently sponsored by the JuS‐Foundation and the Jean Uhrmacher Foundation. Christoph Arolt is currently sponsored by the Gusyk Programme of the University Hospital of Cologne.

## Ethics Statement

Ethical approval was not required for this study as it involved the analysis of previously published data, and all original studies had obtained the necessary ethical approvals. This article does not contain any studies with animals performed by any of the authors.

## Consent

Consent for participation was obtained from all individual participants included in this study including explicit consent for the retrospective analysis of the obtained patient samples (no identifying information about participants is available in this article).

## Conflicts of Interest

The authors declare no conflicts of interest.

## Data Availability

The datasets and code generated and analyzed during the current study are available from the corresponding author on reasonable request.
